# The Role of Senescent CD8^+^T Cells in the Pathogenesis of Disseminated Leishmaniasis

**DOI:** 10.3390/pathogens13060460

**Published:** 2024-05-29

**Authors:** Cayo A. Abreu, Maurício Teixeira Nascimento, Olívia Bacellar, Lucas Pedreira Carvalho, Edgar Marcelino Carvalho, Thiago Marconi Cardoso

**Affiliations:** 1 LAPEC-Fiocruz, Salvador 40296-710, Brazil; bmdcayo@gmail.com (C.A.A.); mauriciotnascimento@hotmail.com (M.T.N.); carvalholp76@gmail.com (L.P.C.); imuno@ufba.br (E.M.C.); 2 Immunology Service, Federal University of Bahia, Salvador 40110-060, Brazil; olivinhaufba@gmail.com

**Keywords:** disseminated, leishmaniasis, CD8^+^T cells, senescent

## Abstract

Disseminated leishmaniasis (DL) caused by *L. braziliensis* is characterized by the presence of 10 to more than 1000 lesions spread on the body. While protection against *Leishmania* is mediated by macrophages upon activation by IFN-γ produced by CD4^+^T cells, the pathology of disseminated leishmaniasis (DL) could be mediated by macrophages, NK, and CD8^+^T cells. Herein, we evaluate the participation of senescent CD8^+^T cells in the pathogenesis of DL. **Methods:** Peripheral blood mononuclear cells (PBMCs), biopsies, co-cultures of CD8^+^T cells with uninfected and infected macrophages (MØ), and PBMC cultures stimulated with soluble *L. braziliensis* antigen (SLA) for 72 h from patients with cutaneous leishmaniasis (CL) and DL were used to characterize senescent CD8^+^T cells. Statistical analysis was performed using the Mann–Whitney and Kruskal–Wallis tests, followed by Dunn’s. **Results:** Patients with DL have an increase in the frequency of circulating CD8^+^T cells that present a memory/senescent phenotype, while lesions from DL patients have an increase in the frequency of infiltrating CD8^+^T cells with a senescent/degranulation phenotype. In addition, after specific stimuli, DL patients’ circulating CD8^+^T with memory/senescent profile, showing degranulation characteristics, increased upon SLA stimuli, and those specific CD8^+^T cells from DL patients had an increased degranulation phenotype, causing more apoptosis of infected target cells. **Conclusions:** DL patients show a higher frequency of cytotoxic senescent CD8^+^T cells compared to CL patients, and that could promote the lysis of infected cells, although without parasite killing, releasing *Leishmania* to the extracellular compartment, contributing to the spread of parasites.

## 1. Introduction

American tegumentary leishmaniasis is predominantly caused by *Leishmania* (Viannia) braziliensis, a parasite that is associated with different clinical forms of disease, such as cutaneous, mucosal, and disseminated leishmaniasis (DL) [1,2].

Cutaneous leishmaniasis (CL) is characterized by one or a few round-shaped ulcers starting as small papules in the presence or absence of lymphadenopathy, and they progress into one or more skin ulcers, characterized by a chronic inflammatory response [3,4]. CL is the most prevalent clinical manifestation, and eight countries, including Brazil, contribute 90% of cases [5].

Mucosal leishmaniasis (ML) is at the hyperresponsive spectrum of clinical diseases caused by *L. braziliensis* [6]. Uncontrolled immune responses have been implicated in ML pathogenesis because T cells from ML patients initiate intense responses (characterized by lymphoproliferation and cytokine production) despite the low number of parasites in mucosal lesions [7].

DL is characterized by 10 or more acneiform, papular, and ulcerated lesions spread over at least two areas of the body [8]. There are several indicators of relevance to studying this atypical presentation of tegumentary leishmaniasis. The prevalence of DL increased 20-fold from 1986 to 2012, characterizing it as an emergent clinical form of leishmaniasis caused by *L. braziliensis* [9]; up to 40% of DL patients have mucosal leishmaniasis (ML) [10].

DL has been confounded with diffuse cutaneous leishmaniasis but is quite distinct, as lymphocytes from patients with DL present a Th1 immune response upon stimulation with *Leishmania* antigen [11]. Both parasites and host factors participate in the pathogenesis of leishmaniasis, and we have shown that there are genotypic differences among isolates of *L. braziliensis* that cause DL and CL [12]. Moreover, parasites from DL have more ability to proliferate in monocytes from both CL and DL patients than isolates from CL, although monocytes from DL are more permissive than cells from CL to parasite multiplication [13]

CD8^+^T cells induce an exaggerated inflammatory reaction and tissue damage correlated to granzyme A (GzA) expression in CL [14]. Besides that, it is known that degranulation of CD8^+^CD107a^+^T cells contains intracellular granzyme B (GzB) and perforin, which are crucial to killing infected cells, and GzB promotes cytolysis even in the absence of perforin and consequently the death of infected target cells [15]. Moreover, CD8^+^T cells can induce cytolysis and apoptosis of murine target cells infected with *Leishmania*, but do not kill intracellular amastigotes [16,17].

CD8^+^CD57^+^T cells are defined by some peculiar characteristics: they are antigen-specific, highly oligoclonally expanded, terminally differentiated, senescent, functionally competent memory/effector T lymphocytes that have undergone many rounds of cell divisions [18,19,20,21]. The senescent profile of CD8^+^CD57^+^T cells is associated with chronic antigen stimulation and high cytotoxic activity [22]. Recently, it was observed that CD8^+^CD57^+^T cells express cutaneous lymphocyte-associated antigen (CLA) and were associated with tissue damage observed in CL caused by *L. braziliensis* [23]. CD8^+^T cells expressing GzB were found on CL and DL biopsies, showing their involvement in both clinical forms of the disease [24]. Recently, Milling S reported that senescent CD8^+^T cells are apparently the most important cell population mediating the disease pathology observed in CL [25]. In the present study, we evaluated the participation of senescent CD8^+^T cells in the pathogenesis of DL compared to CL on peripheral blood, biopsies, stimulated in vitro cultures, and performed co-culture functional assays.

## 2. Materials and Methods

### 2.1. Study Design

This is a cross-sectional study aimed at comparing the CD8^+^T cells with senescent profiles from peripheral blood mononuclear cells (PBMCs) ex vivo and after in vitro stimulation with specific stimuli (soluble *L. braziliensis* antigen, SLA), as well as in CD8^+^T cell co-cultures with infected macrophages (MØ) from DL and CL patients. We also characterized phenotypically CD8^+^T cells in biopsied tissues from CL and DL. The study design consists of the following different types of experiments: (1) to determine the phenotypic profile of CD8^+^T cells (ex vivo) in PBMCs from DL and CL patients; (2) to characterize the phenotypic profile of CD8^+^T cells in biopsied tissues from CL and DL; (3) to determine the profile of CD8^+^T cells in PBMCs and production of cytokines in biopsies after stimulation with SLA; and (4) to characterize the phenotypic features of CD8^+^T cells after co-culture with autologous infected MØ.

### 2.2. Study Subjects

This study was performed in Corte de Pedra, an endemic area of tegumentary leishmaniasis with high *L. braziliensis* transmission, located in the southeastern part of Bahia State, Brazil. CL patients had 1–3 typical cutaneous ulcers, and DL patients had more than 10 acneiform, papular, and ulcerated lesions present in at least three distinct parts of the body. Patients with CL (N = 20) had 1 to 3 typical ulcerative skin lesions, and patients with DL (N = 20) had 19 to 678 papular, acneiform, and ulcerated lesions in at least two different parts of the body. The diagnosis confirmation was performed by the detection of DNA for *L. braziliensis* in biopsied tissues obtained from the skin lesions or by the identification of amastigotes in the biopsied tissues [26]. All patients with CL and DL were evaluated before treatment. ATL patients were treated with N-methyl glucamine antimoniate (SbV^+^), marketed as Glucantime^® (^Sanofi Medley, Campinas, Brazil) at 20 mg/kg/day for 20 days for the CL group or for 30 days for the DL group [8,27]. The study was approved by the ethics committee, and all patients signed informed consent and followed the guidelines of the Ethical Committee of the Federal University of Bahia (CAAE: 62974916.8.0000.5577).

### 2.3. PBMC Isolation

Heparinized blood samples from CL patients and DL patients were overlaid in a Ficoll-Hypaque gradient (GE Healthcare, Uppsala, Sweden), and PBMCs were collected and washed twice with PBS 1× at 300× *g* for 10 min. A representative portion of these cells were labeled with APC-conjugated mAb α-CD14 (clone 61D3) (Sigma, St. Louis, MO, USA). Flow cytometry was employed to determine monocyte frequency in each sample adjusted to 5 × 10^4^ events (FACS Canto II flow cytometer, Becton Dickinson and Company, Franklin Lakes, NJ, USA) and analyzed using the FlowJo 7.6.5 program to determine the CD14^+^ quantification to posteriorly differentiate macrophages at the same proportions. Then, the remainder of the PBMC were suspended in 10 mL of PBS 1×, setting the cell concentration to be adjusted for multiple cell assays.

### 2.4. Flow Cytometric Analysis

#### 2.4.1. Peripheral Blood CD8^+^T Cells

To determine the frequencies and cellular phenotype of CD8^+^T cells from total PBMCs, cells were stained using PE-Cy7 conjugated anti-human CD8 monoclonal antibody (clone SK1); FITC-conjugated mouse anti-human CD45RO monoclonal antibody (clone UCHL1); PE-conjugated anti-human CD107a monoclonal antibody (clone H4A3) (BD Biosciences, San Jose, CA, USA); PerCP-Cy5.5 conjugated anti-human CD57 monoclonal antibody (clone HNK-1); and APC-conjugated anti-human GzB monoclonal antibody (clone QA16A02) (BioLegend, San Diego, CA, USA). The same basic stain protocol was used for biopsies and co-culture analysis. In total, 5 × 10^4^ gated events were collected (FACS Canto II flow cytometer, Becton Dickinson and Company, Franklin Lakes, NJ, USA) and analyzed using the FlowJo 7.6.5 program.

#### 2.4.2. Biopsies

Biopsies (two fragments per patient, one used to diagnosis by PCR and parasite isolation for diagnosis) were collected using a 5 mm punch, and one fragment was split in half using a sterile scalpel blade, weighed (generally 150 mg per half of fragment), and used to cell stain for flow cytometry analysis and biopsies culture. One of those half fragments was meshed using a sterile cell strainer of 40 µm nylon mesh (Thermo Fisher Scientific, Waltham, MA, USA) and washed three times with PBS 1×, and the cells were labeled for flow cytometry analysis. To determine the frequencies and phenotype of CD8^+^T cells that infiltrate biopsies, staining was performed using PE-Cy7-conjugated anti-human CD8 monoclonal antibody (clone SK1), FITC-conjugated mouse anti-human CD57 monoclonal antibody (clone NK1), PE-conjugated anti-human CD107a monoclonal antibody (clone H4A3) (BD Biosciences, San Jose, CA, USA), and APC-conjugated anti-human GzB monoclonal antibody (clone QA16A02) (BioLegend, San Diego, CA, USA). The other half of the biopsies from both DL and CL patients were cultured for GzB production quantification.

#### 2.4.3. PBMC Culture

PBMCs (3 × 10^6^ cells) were cultured in polypropylene plates in 1 mL of RPMI medium supplemented with 2 mM L-glutamine, 1 mM sodium pyruvate, 100 U/mL penicillin, 100 mg/mL streptomycin, 0.1 mM non-essential amino acids, and 50 μM β-mercaptoethanol (complete RPMI medium) at 37 °C and 5% CO_2_. Cells were cultured under SLA stimulation (5 μg/mL) and without stimulation for 72 h following the previous protocol described [28]. To determine the frequencies of CD8^+^T cells expressing CD45RO, CD107a, and GzB, PBMCs were stained with PerCP-Cy5.5-conjugated anti-human mAb CD3 (clone UCHT1), PE-Cy7-conjugated anti-human mAb CD8 (clone SK1), FITC-conjugated anti-human mAb CD45RO (clone UCHL1), PE-conjugated anti-human mAb CD107a (clone H4A3) (BD Biosciences, San Jose, CA, USA), and APC-conjugated anti-human GzB monoclonal antibody (clone QA16A02) (BioLegend, San Diego, CA, USA). In addition, the apoptotic cells were determined using the Annexin V/propidium iodate (PI) apoptosis kit (BD Pharmingen^TM^ FITC Annexin V Apoptosis Detection Kit I, San Diego, CA, USA). In total, 1 × 10^5^ gated events from each sample were collected in a FACS Canto II flow cytometer (Becton Dickinson and Company, Franklin Lakes, NJ, USA) and analyzed using the FlowJo 7.6.5 program.

### 2.5. CD8^+^T Functional Assay

#### 2.5.1. MØ Differentiation

To perform macrophage (MØ) differentiation and posterior infection with *L. braziliensis* promastigotes, PBMCs from DL and CL were (5 × 10^7^) incubated at 37 °C with 5% CO_2_ and 2 mL/well of incomplete RPMI in 24-well plates for 2 h to achieve monocyte differentiation by adhesion. Cell cultures were rinsed twice with incomplete RPMI medium to remove the non-adherent cells and maintained in culture with replaced complete RPMI-1640 (supplemented with 10% heat-inactivated fetal calf serum, 100 U/mL penicillin, 100 mg/mL streptomycin, and 2 mL glutamine; Invitrogen, Carlsbad, CA, USA). Media were replaced after 48 h of culture over 7 days. MØ cells from DL and CL patients were infected with stationary growth phase promastigotes of *L. braziliensis* in the proportion of 5 promastigotes per cell. MØ cells from CL were infected with the CL strain, and cells from DL were infected with the DL strain, respectively. Non-ingested promastigotes were washed away with complete RPMI media. The evaluation of infection was performed by cytometer and by microscopic evaluation of circular glass slides on the bottom of witness well preparation, stained with Giemsa. No parasites were found outside the target cells (MØ) after being washed twice with PBS 1×.

#### 2.5.2. CD8^+^T Cells Sorting

Non-adherent PBMCs from the differentiation of monocytes onto macrophages were collected, washed twice with PBS 1× at 300× *g* for 10 min, and frozen at −80 °C for further isolation. CD8^+^T cells were isolated from 1 × 10^8^ PBMC/mL with a magnetic bead sorting system, obtaining untouched CD8 cells according to the manufacturer’s instructions (Untouched CD8^+^T Isolation Kit II Human MACS, Miltenyi Biotec Inc., Auburn, CA, USA). The process was performed twice, and CD8^+^T cells were washed twice with PBS 1× and adjusted at 5 × 10^6^ cells/mL in complete RPMI before being co-cultured with autologous MØ under the conditions previously mentioned. The overall purity (including DL and CL patients) after untouched CD8^+^T cell sorting was 92 ± 2% from non-adherent frozen cells. The overall viability was 85 ± 5%, as determined by trypan blue exclusion.

#### 2.5.3. Co-Cultures

Briefly, uninfected or infected MØ previously placed on a 6-well culture plate were co-cultured with CD8^+^T cells at a 5:1 ratio of effector/target cells at 37 °C and 5% CO_2_ at 48 h as previous described [29]. Co-cultures were allocated into four subgroups: (1) MØ cultured with media only (MØ), (2) *L. braziliensis*-infected MØ (iMØ), (3) uninfected (MØ plus CD8^+^), and (4) *Leishmania*-infected MØ plus CD8^+^ (iMØ plus CD8^+^). After 48 h, the supernatants were collected for cytokine measurement, and the cells were harvested from plates using PBS 1× supplemented with 2 mM of EDTA at 4 °C. Collected cells were washed twice with PBS 1× 300× *g* for 10 min and stained to access CD8 profiles by flow cytometry using PE-Cy7-conjugated anti-human CD8 mAb (clone SK1), FITC-conjugated mouse anti-human CD45RO mAb (clone UCHL1), PE-conjugated anti-human CD107a mAb (clone H4A3) (BD Biosciences, San Jose, CA, USA), and APC-conjugated anti-human GzB mAb (clone QA16A02) (BioLegend, San Diego, CA, USA). In addition, to measure the effects of CD8^+^T cells and parasites, an apoptotic assay was performed using the same co-culture conditions. MØ cells were labeled with APC-conjugated mAb α-CD14 (clone 61D3) (Sigma, St. Louis, MO, USA) and the Annexin V/PI apoptosis kit (BD Pharmingen^TM^ FITC Annexin V Apoptosis Detection Kit I, San Diego, CA, USA). In total, 1 × 10^5^ gated events from each sample were collected in a FACS Canto II flow cytometer (Becton Dickinson and Company, Franklin Lakes, NJ, USA) and analyzed using the FlowJo 7.6.5 program.

### 2.6. Granzyme B Production

The other half of the biopsies from both DL and CL patients (approximately 150 mg per half fragment) were cultured in polypropylene tubes with 1 mL of complete RPMI at 37 °C and 5% CO_2_ for 72 h [30], and the supernatants were collected and frozen at −80 °C to posteriorly measure GzB production. Supernatants from PBMC, biopsies cultures, and co-cultures from CL and DL patients were collected to determine GzB by ELISA (DuoSet^®^ ELISA Development System R&D Systems, Minneapolis, MN, USA), as previously described [31].

### 2.7. Statistical Analysis

A Mann–Whitney U test was used to compare the frequencies of CD8^+^T cell sub-population profiles on peripheral blood, ex vivo biopsies, PBMC cultures, and biopsy cultures from DL and CL patients. Kruskal–Wallis post-testing by Dunn’s was used to analyze the results of CD8^+^T cell sub-population profiles after co-cultures with autologous uninfected and infected macrophages. ELISA quantifications were analyzed using the Mann–Whitney U test. All analyses had cut-offs for statistical significance set at a *p*-value of ≤0.05 and were performed using GraphPad Prism 8.0 (GraphPad Software, San Diego, CA, USA).

## 3. Results

### 3.1. Patients with DL Have an Increase in the Frequency of Circulating CD8^+^T Cells That Present a Memory/Senescent Phenotype

**Figure 1:**
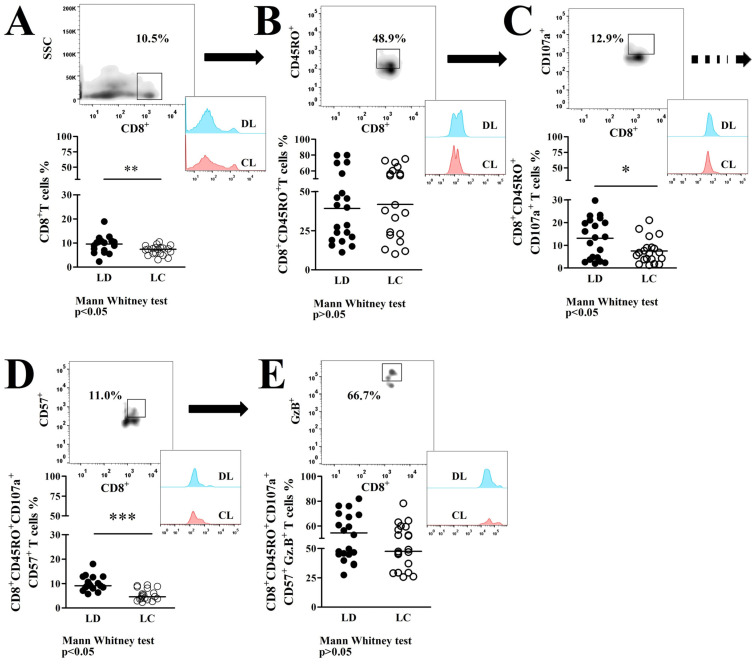
Phenotypic characterization of CD8^+^T cells from the peripheral blood of DL (●) and CL (○) patients: (**A**) In the high left is an SSC vs. CD8 dot plot representing the frequency of total CD8^+^T cells in peripheral blood. Histograms show the frequencies of cell populations in both patient groups. Representative data from the FACS analysis are shown on a graphic in the lower left of the figure. (**B**) Following the same strategy previously described, the frequency of CD8^+^T memory profile cells (CD8^+^CD45RO^+^) was gated from the total CD8^+^T cell population of both groups. (**C**) Frequency of memory CD8^+^T cells presenting cytotoxic characteristics (CD107a^+^) in patients with DL compared to CL. (**D**) Frequency of memory CD8^+^CD45RO^+^CD107a^+^T cells expressing CD57^+^ in the peripheral blood of DL compared to CL samples. (**E**) Frequency of CD8^+^CD45RO^+^CD107a^+^CD57^+^T cells expressing GzB^+^ in the peripheral blood of DL and CL patients (*p* > 0.05). The frequencies are expressed as the median ± SD tested by the Mann–Whitey U test at a *p* ≤ 0.05 significance level. * *p* < 0.05 indicates moderate significance; ** *p* < 0.01 indicates strong significance; and *** *p* < 0.001 indicates very strong significance.

The frequency of peripheral blood CD8^+^T cells in DL patients (9.6 ± 3.4%) was higher (*p* < 0.05) compared to CL (7.0 ± 2.0%), as shown here in Figure 1A. Besides that, the frequency of CD8^+^T cells characterized as having a memory profile (CD8^+^CD45RO^+^) was similar in the DL (39.1 ± 22.4%) and CL (41.8 ± 21.7%) samples (*p* > 0.05; Figure 1B). From that memory cell cluster, a selection of CD8^+^T cells with a degranulation profile (CD8^+^CD45RO^+^CD107^+^) showed a higher frequency of those cells in DL (13.1 ± 8.8%) compared to CL (7.5 ± 5.5%) (*p* ≤ 0.05), represented in Figure 1C. In addition, we showed that from the CD8^+^T cell sub-set, characterized as having a degranulation profile, the frequency of the cells expressing the senescence marker (CD8^+^CD45RO^+^CD107a^+^CD57^+^) in patients with DL (10 ± 2.9%) was higher than in CL patients (5.4 ± 2.3%) (*p* ≤ 0.05; Figure 1D). Regarding that CD8^+^T cell subset with a degranulation-senescence profile, we also evaluated the frequency of those cells expressing GzB with similar results in both groups (34.5 ± 15.2% to DL and 47.5 ± 14.5% to CL), as shown here in Figure 1E (*p* > 0.05).

### 3.2. Ulcers from DL Patients Have an Increase in the Frequency of Infiltrating CD8^+^T Cells with Senescent/Degranulation Phetotype (CD8^+^CD57^+^CD107a^+^)

**Figure 2:**
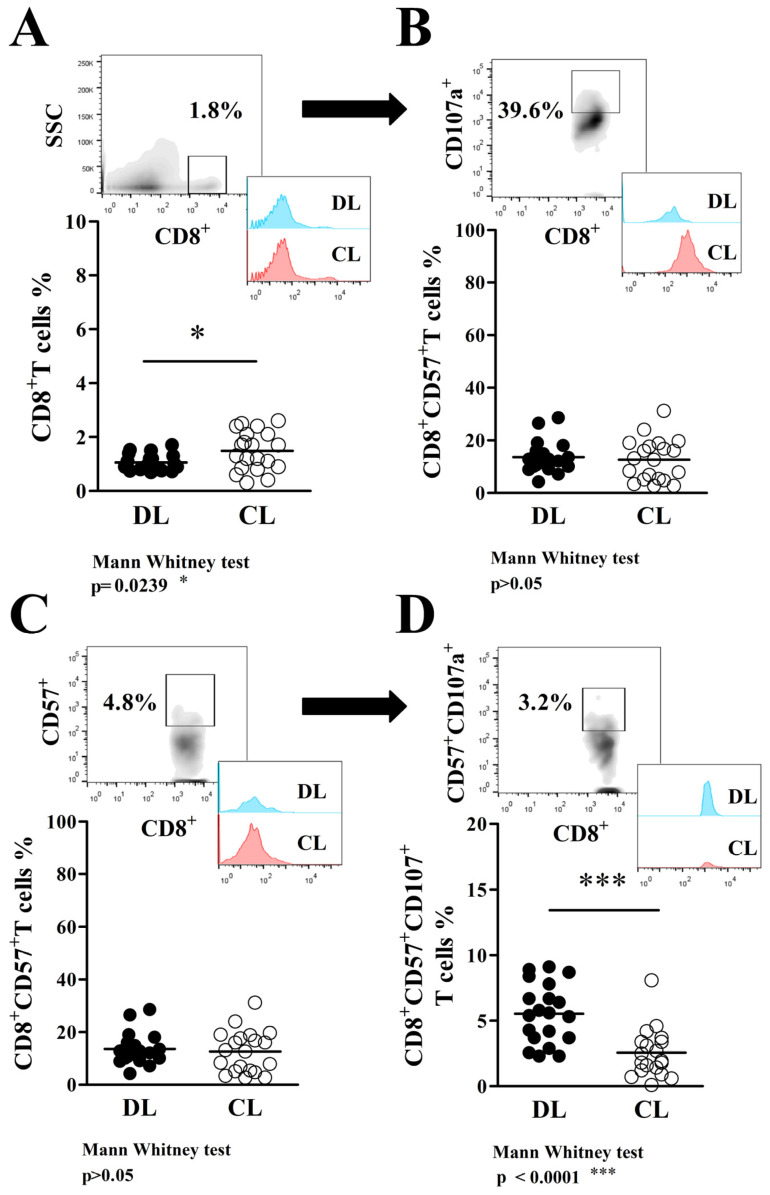
Phenotypic characterization of CD8^+^T cells with senescence/degranulation profile (CD8^+^CD57^+^CD107a^+^) in biopsies of DL (●) and CL (○) patients: (**A**) In the high left is an SSC vs. CD8 dot plot representing the frequency of total CD8^+^T cells in biopsies. Histograms show the frequencies of cell populations in both patient groups. Representative data from the FACS analysis are shown on a graphic in the lower left of the figure. (**B**) Following the same strategy previously described, the frequency of CD8^+^T cells with degranulation characteristics (CD8^+^CD107a^+^) was gated from total CD8^+^T cells on DL and CL biopsies. (**C**) Frequency of cells with a senescence profile (CD57^+^), gated from CD8^+^T cells, on DL and CL biopsies. (**D**) Frequency of CD8^+^T cells with a highly degranulation profile (expressing CD57 and CD107a) observed on DL compared to CL biopsies. The graphic data expressed here are the median ± SD, tested by the Mann–Whitney U test at a *p* ≤ 0.05 significance level. * *p* < 0.05 indicates moderate significance and *** *p* < 0.001 indicates very strong significance.

Differently from what was observed in PBMCs, the frequency of CD8^+^T cells in biopsies of DL patients (1.0 ± 0.3%) was lower than in CL (1.5 ± 0.7%, *p* < 0.05; shown here in Figure 2A), and we also did not find differences (*p* > 0.05) in the frequency of CD8^+^CD107a^+^T cells in DL (40.3 ± 15.0%) and CL biopsies (46.5 ± 17.3%) (data shown in Figure 2B). However, while the frequency of senescence CD8^+^CD57^+^T cells in DL (13.6 ± 5.9%) was similar to CL (12.6 ± 7.9%) (Figure 2C), biopsies from DL patients (5.5 ± 2.3%) showed a higher frequency of senescence CD8^+^T cells with a degranulation profile (CD8^+^CD57^+^CD107a^+^) compared to CL biopsies (2.6 ± 1.8%), as represented here in Figure 2D (*p* < 0.05).

### 3.3. DL Patients’ Circulating CD8^+^T Having a Senescent Profile Showed Intracellular GzB Increased by Specific Stimuli

**Figure 3:**
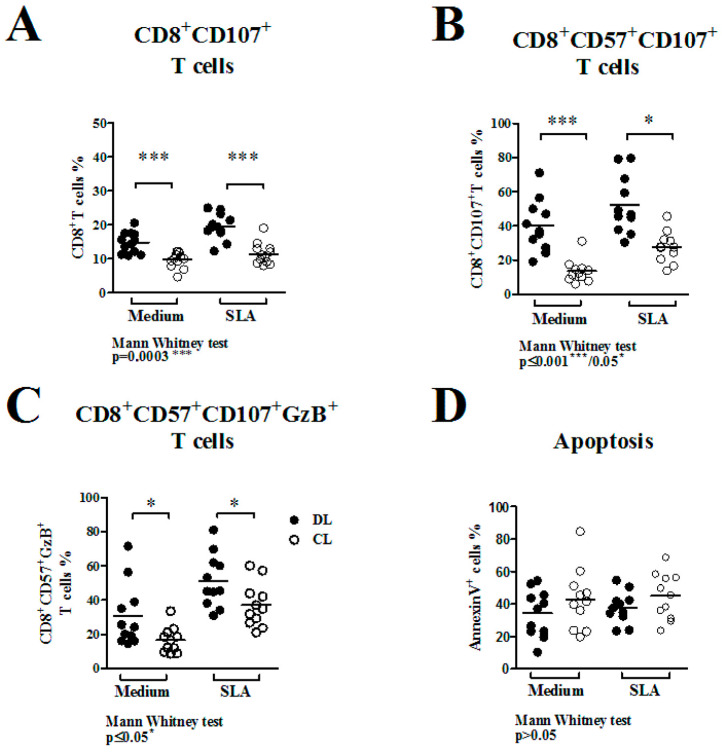
Phenotypic characterization of CD8^+^T cells from PBMC cell culture stimulated with SLA (5 ng/mL) at 72 h. Senescence/degranulation CD8^+^T cell profiles evaluated from DL (●) and CL (○) cell cultures: (**A**) Frequency of degranulation profile CD8^+^T cells (expressing CD107a^+^) from unstimulated (medium) and stimulated (SLA) cell cultures. Representative data from the FACS analysis are shown in the graphic. (**B**) Frequency of CD8^+^T cells with degranulation and senescence characteristics (CD8^+^CD57^+^CD107a^+^) gated from total CD8^+^CD107a^+^T cells on DL and CL cultures. (**C**) Frequency of cells with a degranulation or senescence profile expressing GzB, gated from CD8^+^CD57^+^CD107a^+^T cells. (**D**) Frequency of total apoptotic cells from DL and CL cultures accessed by Annexin V stained and evaluated by FACS. The graphic data expressed here are the median ± SD, tested by the Mann–Whitney U test at a *p* ≤ 0.05 significance level. * *p* < 0.05 indicates moderate significance and *** *p* < 0.001 indicates very strong significance.

The frequency of CD8^+^CD107a^+^T cells from PBMC cultures, unstimulated and stimulated with SLA, showed similar results, with no significant differences when comparing DL and CL patients’ cultures (Figure 3A), revealing an increase in the frequency of antigen-specific CD8^+^CD107a^+^ cells observed in both DL (from 14.7 ± 3.0% to 19.4 ± 3.8% under SLA stimulation) and CL cultures (from 9.6 ± 2.3% to 11.4 ± 3.2%), although there is no difference between the frequencies of this profile of cells after stimulation in both groups (shown here in Figure 3B; *p* > 0.05). For CD8^+^T cells with a senescence/degranulation profile (CD8^+^CD57^+^CD107a^+^), it was observed that there was an increase (although there was no difference between DL and CL) in the frequency after stimulation with SLA (40 ± 15.3%) compared with media only (52.3 ± 16.9%) in DL and CL cultures (13.5 ± 6.7% to SLA and 27.7 ± 9.1% to media only; *p* > 0.05; data shown in Figure 3B). Similar results were observed in terms of the frequency of CD8^+^CD57^+^CD107a^+^ expressing GzB, with an increase in the values before (30.7 ± 18.5% to DL and 16.6 ± 7.5% to CL) and after SLA stimuli (51.3 ± 15.5% to DL and 15.5 ± 12.8% to CL cultures), as shown in Figure 3C (*p* > 0.05). We also measured the apoptosis of total cells before and after stimulation of PBMCs with SLA, and the frequencies of apoptotic cells (Figure 3D) were similar in the DL and CL groups, ranging from 34.1 ± 14.5% to 37.8 ± 9.7% for DL and from 42.9 ± 18.7% to 44.8 ± 14.2% for CL cultures (*p* > 0.05).

### 3.4. Specific CD8^+^T Cells from DL Patients Have an Increased Degranulation Phenotype, Causing More Apoptosis of Infected Target Cells

**Figure 4:**
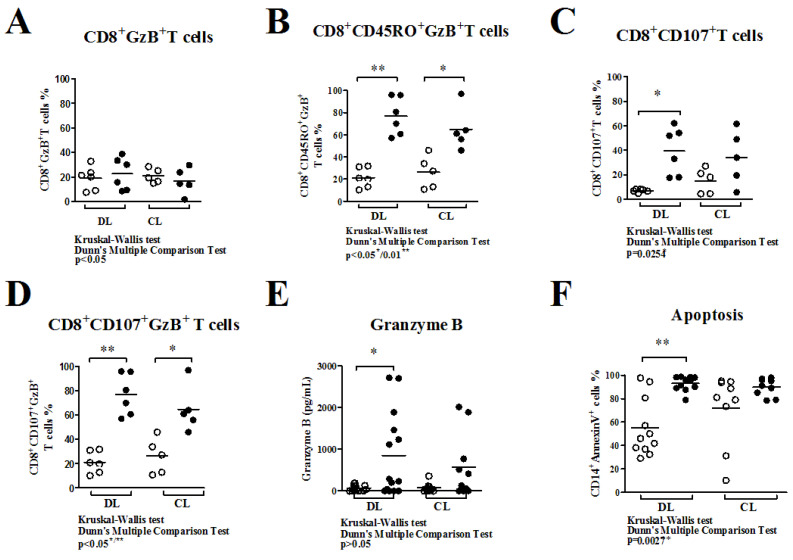
CD8^+^T cell profile, GzB production, and frequency of apoptotic target cells after co-cultures of CD8^+^T cells with uninfected (MØ ○) or infected macrophages (iMØ ●) with *L. braziliensis* (*L.b*). Ratio of 1:5 MØ:*Lb*: (**A**) Frequency of CD8^+^GzB^+^ T cells from DL and CL after 48 h of co-culture with MØ and iMØ. (**B**) CD8^+^CD45RO^+^T cells expressing GzB from both DL and CL co-cultures show a similar profile. (**C**) Frequency of CD8^+^T cells with degranulation characteristics (CD8^+^CD107a^+^) and (**D**) frequency of CD8^+^CD107a^+^GzB^+^ T cells in co-cultures. (**E**) GzB production in the supernatants of CD8^+^T cell co-cultures. (**F**) Frequency of apoptotic target cells (CD14^+^AnnexinV^+^) in co-cultures of DL and CL patients. Kruskal–Wallis U test. *p* ≤ 0.05 was a significant level. * *p* < 0.05 indicates moderate significance and ** *p* < 0.01 indicates strong significance.

The frequency of CD8^+^T cells expressing GzB was similar in co-cultures with uninfected macrophages (MØ) (19.0 ± 9.5% to DL and 20.8 ± 5.7%) and infected macrophages (iMØ) (22.6 ± 13.1% to DL and 16.7 ± 10.7%), as shown in Figure 4A. After co-culture, the frequency of CD8^+^CD45RO^+^T cells expressing GzB increased (*p* < 0.05 and *p* ˂ 0.01) in co-culture with iMØ compared to MØ co-cultures from DL (from 21.0 ± 8.9% to 78.5 ± 15.6%) and CL patients (from 28 ± 18.1% to 62.7 ± 25.6%), as shown here in Figure 4B. Moreover, while in the iMØ co-culture with autologous CD8^+^T cells from DL patients there was an enhancement in the frequency of CD8^+^CD107a^+^T cells (39.4 ± 19.3%), in comparison with MØ co-culture (7.1 ± 1.3%), this was not observed in the iMØ co-culture of CL patients (15.0 ± 10.1%) compared to MØ (33.9 ± 12.3%) co-cultures (Figure 4C). CD8^+^T cells expressing GzB showed a degranulation profile (CD107a^+^), and both DL and CL co-cultures had an increase in the frequency of those cells, comparing MØ (21.1 ± 9.0% to DL and 26.2 ± 14.7% to CL) to iMØ (76.7 ± 17.0% to DL and 64.8 ± 19.2%) co-cultures (Figure 4D). However, while there was a significant increase in the production of GzB in supernatants of iMØ plus CD8^+^T cells co-cultures from DL (from 67 ± 70.5 pg/mL to 849.4 ± 1009 pg/mL; *p* < 0.05), there was no difference in the production of GzB in co-cultures of iMØ plus CD8^+^T cells (576.2 ± 769.6 pg/mL) versus MØ plus CD8^+^T cells co-cultures (72.2 ± 107.5 pg/mL) from CL patients (Figure 4E; *p* > 0.05). Indeed, while an enhancement in the frequency of apoptotic target cells was observed in co-cultures of iMØ plus CD8^+^T cells (96 ± 18.7%) in comparison with co-cultures MØ plus CD8^+^T cells (58 ± 35%) from DL patients, there was no significant enhancement in the frequency of apoptotic target cells observed in MØ plus CD8^+^T cells (75 ± 45%) compared to that observed in iMØ plus CD8^+^T cells co-cultures (94 ± 17%) from CL patients (Figure 4F; *p* > 0.05. 35%). In DL patients, there was no significant enhancement in the frequency of apoptotic target cells observed in MØ plus CD8^+^T cells (75 ± 45%) compared to that observed in iMØ plus CD8^+^T cells co-cultures (94 ± 17%) from CL patients (Figure 4F) *p* > 0.05.

## 4. Discussion

CD8^+^T cells are related to tissue damage in tegumentary leishmaniasis, contributing to the enhancement of the inflammatory response in *L. braziliensis* infection [32]. This has also been well documented in the tissues of CL patients [14,33,34]. Pathology in CL has been associated with activation of CD4, CD8, and NK cells [35]; moreover, transcriptomic analysis of skin lesions from CL reveals a strong CD8^+^ T cell immunosenescence signature linked to the immunopathology of that clinical form [36].

There is an increase in the intensity of the inflammatory reaction from early CL to classical CL, and this inflammatory reaction is associated with an increased frequency of CD8^+^T cells expressing granzyme A during the progression of the disease [23]. It has been shown that CD4^+^T cells expressing IFN-γ or TNF, as well as CD4^+^ and CD8^+^T cells expressing CD107a, are associated with tissue damage through lymphocyte extracellular traps (LETs) and vesicles expressing CD107a [37].

These studies provided strong evidence for the participation of CD8^+^T cells in the pathogenesis of CL; however, the participation of CD8^+^T cells, with their different profiles, in DL pathology, contributing to the development of usually multiple ulcers, is not well understood. Herein, it was evaluated which CD8^+^T cell subset participates in the pathology of DL and its effects, involving cytotoxic activity, correlated with tissue scattering damage and parasite dissemination. The cytotoxicity activity of CD8^+^T cells was also emphasized by the documentation of an increased frequency of CD8^+^CD107a^+^T cells [31] and effector memory/senescent CD8^+^CD57^+^T cells in peripheral blood and biopsies of CL patients [23,25,38]. Moreover, CD8^+^CD57^+^T cells were also enhanced in patients with active CL in comparison with both cured CL and subjects with subclinical *L. braziliensis* infection [39]. Herein, it was shown that DL patients have an increased frequency in peripheral blood of total CD8^+^T cells with degranulation (CD8^+^CD107a^+^) and senescence (CD8^+^CD57^+^CD107a^+^) profiles compared to CL patients.

Circulating memory/senescence CD8^+^T cells were associated with systemic inflammation and larger lesion size during active CL caused by *L. braziliensis* [40]. These data demonstrate that differences in memory and senescent CD8^+^CD57^+^T cells observed in DL could drive them to the lesion site and lead, by cytotoxicity, to more tissue damage compared to CL patients. Likewise, as shown in peripheral blood, skin biopsies from DL patients show an increase in the frequency of infiltrating CD8^+^T cells with a senescent/degranulation phenotype (CD8^+^CD57^+^CD107a^+^), corroborating the hypothesis that cells could drive the lysis of infected cells, leading to tissue destruction, the development of skin lesions, and, furthermore, parasites releasing intracellular viable parasites from infected cells.

Some authors related that T cells expressing CD57^+^ were shown in high frequency on CL lesions caused by *L. braziliensis* [21]. Corroborated with our hypothesis, Milling S. showed that in infected tissues, CD8^+^CD57^+^T cells are responsible for the high cytotoxicity observed in CL skin lesions and higher damage capability compared to infiltrated NK cells expressing CD57^+^ [25]. In fact, we find the same profile of NK cells in DL patients with less capability compared to CD8^+^CD57^+^T cells in both DL and CL ulcers.

As it has been suggested that the senescence CD8^+^T cells have a decreased migration mobility capacity once in the tissue [23], this may explain the higher proportion of degranulating CD8^+^CD57^+^T cells in biopsy than in the blood of DL patients compared to CL patients. Alternatively, CD8^+^T cells, once in the tissue, can acquire the CD57^+^ cell marker with a high cytotoxicity capability, even if they present similar intracellular GzB production and secretion observed in DL compared to patients with CL. These data corroborate that CD8^+^CD57^+^CD107a^+^T cells in DL lesions could degranulate more easily compared to CL lesions once in tissue. GzB promotes lysis of the infected cells, releasing the parasites.

Senescence CD8^+^T cells have reduced proliferation capability and high cytotoxic capacity [41], and here we show that, in addition, senescent CD8^+^T cells from DL patients show higher degranulation activity (CD8^+^CD57^+^CD107a^+^) compared to CL patients on biopsies. In addition, the frequency of senescence CD8^+^T cells after in vitro PBMC was evaluated upon specific stimuli.

Although there is evidence that senescent CD8^+^ T cells have a non-specific cytotoxic response in tissue from CL biopsies and directly contribute to tissue damage [40], herein, it was shown that senescent CD8^+^ T cells, in PBMC cultures, stimulated with a specific antigen of *L. braziliensis* (SLA), have low frequencies compared to unstimulated cultures. Nevertheless, there is a higher frequency of CD8^+^ T cells with features of degranulation (CD107a^+^) and senescence CD8^+^CD57^+^CD107a^+^ expressing GzB in unstimulated PBMC cultures and under SLA stimuli in patients with LD compared to patients with CL.

Senescent CD8^+^ T cells were previously higher in patients with DL compared to patients with CL, with an increase after specific stimuli. The equal apoptosis observed in DL and CL cultures (Figure 3D) can be explained by the absence of intracellular parasites, unlike what occurred in co-cultures with CD8^+^ T cells and infected macrophages (Figure 4F).

Previously, it was shown in co-cultures of CD8^+^T cells with uninfected and infected monocytes, while in subclinical *L. braziliensis* infection, that these cells have a greater capacity to kill *L. braziliensis*-infected monocytes than CL CD8^+^T cells, and there was more apoptosis of infected monocytes in co-culture with CD8^+^T cells of CL than with cells of subjects with subclinical infection [32].

In autologous co-cultures with infected macrophages, cells are also responsible for the pronounced apoptosis effect in infected target cells. Autologous CD8^+^T cells from DL or CL patients were co-cultured with autologous uninfected or infected macrophages (a ratio of five CD8^+^T cells per target cell). Compared to co-cultures with uninfected macrophages, there was an enhancement in the frequency of CD8^+^CD107a^+^T cells, followed by higher GzB production and higher apoptosis of infected macrophages in co-cultures from DL patients compared to CL. In co-cultures, CD8^+^CD107a^+^GzB^+^T cells had a higher frequency in DL and CL co-cultures with infected macrophages compared with uninfected cells. While there was no difference in the apoptosis in target cells driven by CD8^+^ in CL patients, there was an increase in apoptotic target cells in co-cultures of infected macrophages in DL patients, which showed more capability to induce the death of host cells, realizing intracellular parasites.

The sum of the findings suggests that, despite having a higher cytotoxic effect in DL patients, senescence CD8^+^T cells could promote lysis of infected cells but are not able to kill intracellular parasites, releasing *Leishmania* amastigotes to the extracellular compartment, promoting parasite metastasis, contributing to the spread of disease, and consequently leading to disseminated disease.

## Data Availability

The original contributions presented in the study are included in the article, further inquiries can be directed to the corresponding author.
